# Effect of Resistance Training With Total and Partial Blood Flow Restriction on Biomarkers of Oxidative Stress and Apoptosis in Untrained Men

**DOI:** 10.3389/fphys.2021.720773

**Published:** 2021-09-09

**Authors:** Fabio Rocha de Lima, Douglas Popp Marin, Letícia Torres Ferreira, Celso Pereira Batista Sousa Filho, Todd Anthony Astorino, Jonato Prestes, Marcelo Luis Marquezi, Rosemari Otton

**Affiliations:** ^1^Interdisciplinary Post-graduate Programme in Health Sciences, Cruzeiro do Sul University, São Paulo, Brazil; ^2^Department of Kinesiology, California State University San Marcos, San Marcos, CA, United States; ^3^Graduation Program on Physical Education, Catholic University of Brasilia, Brazilia, Brazil; ^4^Physical Education Research Laboratory, Universidade Cidade de São Paulo, São Paulo, Brazil

**Keywords:** occlusion training, kaatsu training, strength training, oxidative stress, strength development

## Abstract

**Introduction:** The characterization of immune and oxidative stress responses to acute and chronic exercise training is important because it may aid in the safety and dose–response prescription of resistance training (RT) in many populations.

**Purpose:** The present study compared changes in acute oxidative stress and markers of apoptosis in immune cells before and after 8 weeks of low-load RT with total or partial blood flow restriction (BFR) versus high-load traditional RT.

**Methods:** Twenty-seven untrained men were randomly divided into three groups: traditional RT [75% one-repetition maximum (1-RM)], RT with partial (20% 1-RM), and total BFR (20% 1-RM). Over an 8-week period, participants performed six sets of arm curls until failure with 90 seconds of recovery for 3 days/week. Blood samples were obtained before and after the first and last training sessions.

**Results:** Data indicated that all training groups showed similar increases in muscular strength (*p* < 0.001), reduction in mitochondrial membrane potential (MMP) after exercise in neutrophils (*p* < 0.001), and increase in caspase-3 activity after exercise (*p* < 0.001). Traditional RT and total BFR showed increased plasma lipid peroxidation (*p* < 0.001) and protein carbonyls (*p* < 0.001) and lower levels of reduced glutathione (GSH) (*p* < 0.001) after exercise. No change was observed in oxidative stress biomarkers in response to partial BFR (*p* > 0.05).

**Conclusion:** Data show that RT with partial BFR can increase muscular strength but still does not augment biomarkers of oxidative stress in untrained men. In addition, RT with total BFR promoted similar responses of oxidative stress and markers of immune cell apoptosis versus traditional RT.

## Introduction

Resistance training (RT) increases muscular strength, power, muscle mass, functional capacity, sports performance, and general health (American College of Sports Medicine, 2009). RT intensity is generally considered as one of the most important variables for developing muscular adaptation (American College of Sports Medicine, 2009). It is evident that intensity ≥70% of the individual one-repetition maximum (1-RM) promotes substantial training adaptations (American College of Sports Medicine, 2009), while for clinical populations (e.g., patients undergoing rehabilitation or having underlying disease) and untrained adults, RT with such high loads may be difficult to apply (Clarkson et al., 2020).

Blood flow restriction (BFR) is an emergent training method which uses a cuff placed at the proximal portion of the exercising limb to restrict venous outflow in working musculature (da Cunha Nascimento et al., 2020). Results from meta-analyses in healthy and clinical populations demonstrate that RT combined with BFR using 20–30% 1-RM elicits similar changes in muscular endurance, muscular strength, and hypertrophy versus traditional high-load RT (Slysz et al., 2016; Lixandrao et al., 2018; Centner et al., 2019), and was superior to load-matched RT without BFR in promoting muscle hypertrophy and strength gains (Slysz et al., 2016). Therefore, low-load RT with BFR may be an alternative to traditional high-load RT for untrained subjects and populations with severe physical limitations. In addition to its use in clinical prevention and rehabilitation settings, RT with BFR may be an alternative method to maximize neuromuscular adaptations without increasing joint stress (Lixandrao et al., 2018).

It is evident that the cuff pressure selected during BFR training has the potential to promote different acute (Clarkson et al., 2020) and chronic (Slysz et al., 2016; Clarkson et al., 2020) responses. Although the effects of RT with BFR on muscle strength are well documented (Slysz et al., 2016; Lixandrao et al., 2018; Centner et al., 2019), less is known about the modulating effects of BFR on markers of apoptosis in immune cells and oxidative stress. Apoptosis is a form of programmed cell death, which is an important physiological mechanism to maintain homeostasis in the body (Czabotar et al., 2014). In leukocytes, apoptosis is the central mechanism in the regulation of their life span and to support their cellular function (Elmore, 2007). Two main mechanisms for apoptosis, namely, extrinsic (cell surface death receptor) pathway and intrinsic pathway, which is also called the mitochondrial pathway [reduction of mitochondrial membrane potential (MMP)], have been described (Bock and Tait, 2020). Both pathways converge in the activation of caspase-3, described as an effector or executioner of apoptosis, causing DNA fragmentation as well as degradation of cytoskeletal and nuclear proteins (Elmore, 2007).

Prior studies revealed that the acute response of markers of apoptosis to RT is protocol-dependent (Krüger et al., 2011; Pereira et al., 2012; Prestes et al., 2014). In untrained young men, Prestes et al. (2014) reported a significantly greater increase in markers of apoptosis in CD4+ lymphocytes following RT designed to promote muscle hypertrophy compared to a protocol designed to enhance muscular endurance. Sharafi and Rahimi (2012) examined the acute response of serum caspase-3, marker of apoptosis, immediately, 3 and 24 h following four sets of six whole-body exercises at 80% 1-RM to failure in trained and untrained men. Caspase-3 was significantly higher after exercise in untrained compared to trained men. Although these data suggest a protective mechanism against apoptosis with RT in trained men, no previous study investigated the long-term adaptations of biomarkers of apoptosis to RT with or without BFR. Kruűger et al. (2009) proposed that apoptosis of lymphocytes induced by exercise would be a regulatory mechanism to remove activated and potentially autoreactive immune cells. Moreover, the “immune space theory” indicates that acute exercise might create immune space by removing senescent lymphocytes to undergo apoptosis in the tissues and allow new “recruits” to take their place (Simpson, 2011; Peake et al., 2017).

The reduction of MMP has been extensively used as a biomarker of leukocyte apoptosis (Otton and Curi, 2005; Syu et al., 2011; Bock and Tait, 2020). Previous studies have shown that repeated exhaustive aerobic exercise reduces MMP in neutrophils (Hsu et al., 2002; Tuan et al., 2008). For example, Syu et al. (2011) demonstrated a significant reduction of MMP in neutrophils immediately after an acute bout of aerobic exercise until exhaustion in untrained men, indicating an increase in spontaneous neutrophil apoptosis.

Among the mechanisms explaining exercise-induced cell apoptosis, the oxidative stress-mediated intrinsic pathway (Kruger and Mooren, 2014) occurs when there is an imbalance between oxidants and antioxidants in favor of the oxidants (Fisher-Wellman and Bloomer, 2009). Acute bouts of RT with (Centner et al., 2018) or without BRF (Ismaeel et al., 2019) significantly increase the generation of reactive oxygen species (ROS). Centner et al. (2018) demonstrated that acute lower body RT at 30% 1-RM with partial BFR increased systemic ROS production versus pre-exercise. Therefore, it is possible that chronic exposure to ROS during RT upregulates antioxidant defense systems and subsequently reduces biomarkers of oxidative stress in the blood (Azizbeigi et al., 2013; Ismaeel et al., 2019).

The characterization of immune and oxidative stress responses to acute and chronic exercise training is important because it may optimize the safety and dose–response prescription of RT in many populations (Marin et al., 2013). Thus, the purpose of the present study was to compare changes in acute oxidative stress and markers of apoptosis in immune cells before and after 8 weeks of low-load RT with total or partial BFR versus high-load traditional RT (TRAD). It is hypothesized that low-load RT with total BFR induces a similar acute response on oxidative stress and apoptosis markers versus traditional high-load RT, but this will be higher versus partial BFR. In addition, we expected that the magnitude of acute responses of these biomarkers would be attenuated following 8 weeks of training. In this regard, the upregulation of antioxidant defense systems by RT with BFR may downregulate the markers of apoptosis in leukocytes induced by an acute bout of resistance exercise.

## Materials and Methods

### Experimental Design

**FIGURE 1:**
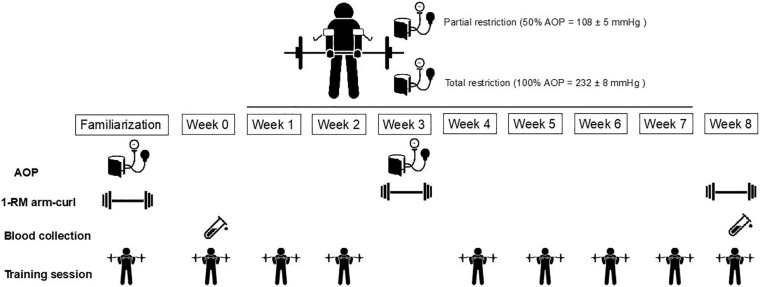
Experimental design. AOP, arterial occlusion pressure.

During the first visit to the laboratory, anthropometric parameters were measured, and all participants completed health questionnaires to determine eligibility. In addition, participants underwent testing of 1-RM and were familiarized with RT with BFR (Figure 1). Changes in parameters representing oxidative stress and apoptosis were measured following the 1st (week 0) and in the 17th (week 8) training sessions. In this study, we measured MMP and caspase-3 as biomarkers of leukocyte apoptosis (Otton and Curi, 2005). The external load and arterial occlusion pressure used during training sessions were assessed and readjusted at week 3. All RT sessions were performed at the same time of day (1–3 p.m.) according to the preferences of participants and time availability. Participants were asked to maintain their regular diet and to refrain from exercise 24 h before each session, and they were instructed to come to each session well rested and hydrated. The participants reported to the laboratory after consuming their habitual diet 1 h before the sessions.

### Participants

Twenty-seven physically active men participated in the study. They habitually completed the aerobic exercise and free-living activity >150 min/week in the last year, as determined by the International Physical Activity Questionnaire (IPAQ) (Craig et al., 2003), yet none was training for any sport. Inclusion criteria included no systematic RT in the previous 6 months, no prior experience in performing an exercise with BFR, and no use of any medications, tobacco, or nutritional supplements. Before testing, participants were randomly allocated to perform traditional RT [TRAD; *n* = 9; age = 22.0 ± 1.9 years; and body mass index (BMI) = 22.3 ± 2.7 kg/m^2^], partial BFR (PR; *n* = 9; age = 23.1 ± 2.2 years; and BMI = 24.2 ± 2.4 kg/m^2^), and total BFR (TR; *n* = 9; age = 23.3 ± 2.0 years; and BMI = and 22.8 ± 2.7 kg/m^2^). The experimental procedures and risks were thoroughly explained, and all volunteers provided written informed consent according to the Declaration of Helsinki of the World Medical Association. This study was approved by the Ethics Committee of Cruzeiro do Sul University under the protocol 64377817.4.0000.8084.

### One-Repetition Maximum Testing and Exercise Volume

Maximal strength was assessed using a concentric 1-RM arm curl test (Body great, Sao Paulo, Brazil) according to the guidelines established by the National Strength and Conditioning Association (McGuigan, 2016). Briefly, participants performed 1 set of 10 repetitions with 50% of the estimated 1-RM. After 2 min of rest, three repetitions were performed with a load equal to 70% of estimated 1-RM. Following 3 min of rest, participants completed three to five 1-RM attempts with progressively heavier weights (+5%), interspersed with 5-min rest intervals until a 1-RM was determined. Proper technique and a complete range of motion were required for the determination of true 1-RM. Verbal encouragement was provided by the researchers throughout testing. Failure to lift the load or lifting with incorrect technique disqualified the attempt. 1-RM was identified as the highest load lifted once with proper form.

### Volume Load

Volume load during RT was calculated from training logs for each participant and was assessed by the equation: sets × repetitions × external load (kg) (Scott et al., 2016). Only repetitions performed through a full range of motion and using proper technique were included for analysis.

### Blood Collection and Cell Separation

In all participants, blood samples from the antecubital vein were obtained before and immediately after the first and last RT sessions. Blood samples (20 ml) were acquired pre-exercise with participants rested in a seated position for 5 min and immediately after the sixth set of RT. The samples were collected in vacutainer tubes containing 0.1 mM ethylenediaminetetraacetic acid (EDTA). The sample was stored on ice for 30 min and Histopaque 1.077 was used for isolation of leukocytes according to the study by Otton and Curi (2005). Freshly isolated lymphocytes and neutrophils were used immediately for measuring MMP.

Blood (∼5 ml) was centrifuged at 1,200 rpm for 10 min at 4°C to obtain plasma. Plasma aliquots (100 μl) were stored at −80°C (Revco, Asheville, NC, United States) until analyzed for the dependent variables. Lymphocytes and neutrophils viability was assessed by using Trypan Blue exclusion in the Neubauer chamber.

### Mitochondrial Membrane Potential and Caspase-3 Activity

The MMP was evaluated using Rhodamine 123 (Rh123) (Thermo Fisher, Eugene, OR, United States) (Otton and Curi, 2005). Five micromolars of Rh123 was added to neutrophils and lymphocytes (5 × 10^5^/well), and carbonyl *m*-chlorophenylhydrazone cyanide (2 μM), an uncoupler of the proton gradient, was used as an internal control of the experiment. Rh123 was monitored at the excitation wavelength of 510 nm and emission of 530 nm in the microplate reader (Tecan, Salzburg, Austria).

The caspase-3 activity of neutrophils and lymphocytes was evaluated using the EnzChek Caspase-3 Assay Kit #2 (Thermo Fisher, Eugene, OR, United States). Briefly, this method evaluates the activity of caspase-3 through the cleavage of the substrate derived from aminomethyl coumarin according to the instructions of manufacturer.

### Measurement of Thiobarbituric Acid Reagent Substances and Contents of Free Thiol Groups and Carbonylated Contents

The thiobarbituric acid reagent substances (TBARS) assay was performed according to the study by Fraga et al. (1988) and detailed in the study by Marin et al. (2011b). Thiol groups were evaluated as biomarkers of the redox state by the presence of free sulfhydryl group. The reduction of the thiol groups was detected by the formation of color adducts after the reaction with 4 mM 5,5′-dithiobis(2-nitrobenzoic acid) (DTNB). The absorbance of DTNB-treated samples was evaluated at 412 nm and calculated using reduced glutathione (GSH) as standard (0–100 μM).

To estimate the carbonylated proteins, the same procedures described above were used. The carbonylated proteins were identified by the hydrazones identified with 10 mM dinitrophenylhydrazine in 0.25 M HCl (Marin et al., 2011a). The absorbance peak detected within the 340–380 nm range was identified and the concentration of the carbonyl groups was calculated based on the molar coefficient of ε = 2.2 × 104 M^–1^ cm^–1^ using serum albumin as the standard (0–1,000 μg/ml).

### Plasma Iron Reduction Ability

The plasma iron reduction ability (FRAP) assay was based on a single electron transfer reaction between antioxidants using Fe^3+^ as the oxidant (Marin et al., 2013). The change in the absorption spectrum of the Fe^2+^ complex, due to the action of an antioxidant in the plasma, was evaluated at 593 nm in a spectrophotometer (Ultrospec 3000, Pharmacia Biotech, Canada). The standard curve was linear between 0 and 100 μM FeSO_4_.

### Determination of GSH and Oxidized Glutathione Content

The method described by Rahman et al. (2006) was used to determine the content of glutathione. For estimating the total values of GSH and oxidized glutathione (GSSG), they were analyzed using DTNB to combine with GSH to form 5-thio-2-nitrobenzoic acid. The concentration of GSH/GSSG was calculated from a standard curve prepared with pure GSH/GSSG (0–50 μM) and expressed as μM GSH and GSSG (Marin et al., 2013).

### Resistance Training Program

The 8-week RT program consisted of six sets of barbell arm curl exercise (Body great, Sao Paulo, Brazil) performed to concentric failure, interspersed with 90s of passive recovery between sets, 3 days/week on non-consecutive days. According to previous studies, 8 weeks of RT with BFR promotes metabolic and neuromuscular adaptations in untrained participants, and allows the observation of possible differences in strength and oxidative stress biomarkers between training conditions (Slysz et al., 2016; Patterson et al., 2019). Men in the TRAD group performed RT at 75% of 1-RM without BFR, while men in PR and TR exercised at 20% of 1-RM with arterial occlusion pressure, as this is a commonly used low-intensity BFR training load (da Silva et al., 2016; Centner et al., 2018; Ismaeel et al., 2019). In the PR and TR groups, arterial occlusion pressure was equal to 50% (108 ± 5 mmHg at week 0 and 107 ± 5 mmHg at week 3) and 100% (232 ± 8 mmHg at week 0 and 233 ± 8 mmHg at week 3). Participants were supervised on correct lifting technique and were instructed to perform repetitions using the full range of motion in a controlled manner with self-selected tempo during concentric and eccentric actions. They were also instructed to perform as many repetitions as possible to failure during each set, and total repetitions were recorded in all sessions. All training sessions started with a standardized warm-up consisting of 15 repetitions of bilateral arm curl at a load equal to 30% 1-RM without BFR. BFR was maintained throughout training and recovery periods in PR and TR, and the cuff was removed only at the end of the session. During the 8-week training regimen, participants did not perform any other type of physical activity which was confirmed with a written log.

To implement BFR, a cuff 6.5 cm wide and 80 cm long (Cardiomed^®^, Sao Paulo, Brazil) was placed over the proximal portion of the arm of each participant. A portable Doppler probe (DF-7001 VN; MEDPEJ; São Paulo, Brazil) was placed over the radial artery until a pulse was detected. The pressure inside the cuff was inflated until a pulse could no longer be detected; the lowest pressure at which occlusion occurred was recorded as the arterial occlusion pressure.

### Statistical Analysis

Data are expressed as mean ± SD and were analyzed using SPSS 22.0 software for Mac (SPSS, Inc., Chicago, IL, United States). Normality of variables was verified using the Shapiro–Wilk test. One-way ANOVA was used to identify differences in baseline variables between groups. A repeated measure analysis of variance (time = 4 levels × group = 3 levels) was used to identify differences in outcome measures in response to training. Assumptions of sphericity were evaluated using the Mauchly test. The Greenhouse–Geisser correction was used to account for the sphericity assumption of unequal variances across groups. For the analysis of GSSG content, the two-way ANCOVA with repeated measures was used with pre-exercise GSSG at baseline as a covariate. Effect sizes were evaluated using a partial eta squared (η^2^_*p*_) for each ANOVA, with 0.06, 0.06–0.14, and >0.14 indicating a small, medium, and large effect, respectively. *Post hoc* comparisons were performed with the Bonferroni correction. For pairwise comparisons, Cohen’s *d* for repeated measures and between groups was calculated. The threshold values for ≥0.2 and <0.5 (small), ≥0.5 and <0.8 (moderate), ≥0.8 and <1.3 (large), and ≥1.3 (very large) were considered according to the study by Rosenthal (1996). The statistical power observed ranged from 0.95 to 1.00 for all variables analyzed, except for the GSSG that showed a lower observed power of 0.77 for the effect of time from pre–post exercise. Statistical significance was set as *p* < 0.05.

## Results

Exercise sessions were well tolerated by participants and all data presented are derived from nine participants in each group. Participants completed 100% of all required sessions of RT.

### Exercise Volume and 1-RM Arm Curl

**FIGURE 2:**
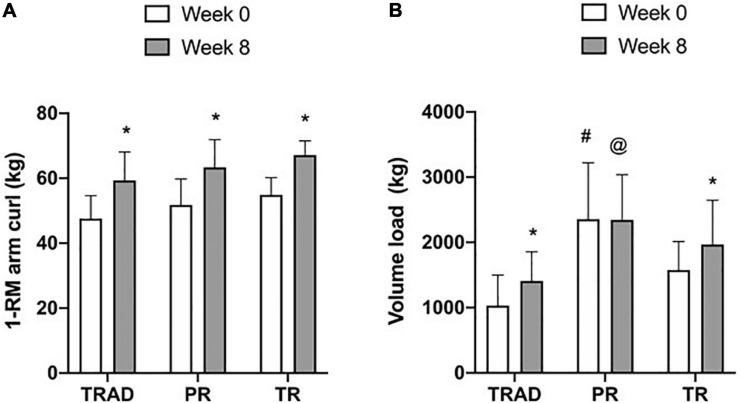
Change in 1-RM arm curl strength **(A)** and training volume **(B)** at week 0 (1st session) and week 8 (17th session). TRAD, traditional resistance training; PR, partial restriction; TR, total restriction. Results are presented as mean ± SD. *p < 0.05 from week 0, ^#^different from TRAD and TR, and ^@^different from TRAD.

Before the RT intervention, there was no difference between groups in 1-RM arm curl (*p* = 0.10). Changes in 1-RM arm curl strength are presented in Figure 2A. Compared to week 0, there was a significant increase in 1-RM strength (*p* < 0.001; η^2^_*p*_ = 0.91), with no difference between groups (*p* = 0.09; η^2^_*p*_ = 0.18) or time × group interaction (*p* = 0.93; η^2^_*p*_ = 0.005). Compared to week 0, arm curl strength increased in TRAD (15%; *d* = 1.74; *p* < 0.001), PR (16%; *d* = 2.19; *p* < 0.001), and TR (14%; *d* = 2.08; *p* < 0.001).

There was an increase in training volume (*p* = 0.009; η^2^_*p*_ = 0.25) with a difference between groups (*p* = 0.001; η^2^_*p*_ = 0.42), but no time × group interaction (*p* = 0.14; η^2^_*p*_ = 0.15) (Figure 2B). At baseline, training volume was greater for the PR when compared with TRAD (*d* = 1.90; *p* < 0.001) and TR (*d* = 1.14; *p* = 0.04). At week 8, training volume was greater for PR when compared with TRAD (*d* = 1.61; *p* = 0.01), but not with TR (*p* = 0.61). Compared to week 0, there was an increase in training volume for adults performing TRAD (*d* = 0.95; *p* = 0.02) and TR (*d* = 1.16; *p* = 0.02), but not for PR (*p* = 0.94).

### Antioxidant Parameters

Table 1 shows the acute responses of FRAP, GSH, GSSG, and GSH/GSSG ratio to the various RT conditions. There was a decrease in FRAP after exercise (*p* < 0.001; η^2^_*p*_ = 0.58) and a significant time × group interaction (*p* = 0.002; η^2^_*p*_ = 0.25), while it was not different among groups (*p* = 0.36; η^2^_*p*_ = 0.08). Compared to pre-exercise, at week 0, FRAP concentrations were decreased after exercise in the TRAD (*d* = 4.70; *p* < 0.001) and TR (*d* = 3.42; *p* < 0.001) groups but not in the PR (*p* = 0.07) group. At week 8, there was a decrease in FRAP after exercise in TRAD (*d* = 2.34; *p* < 0.001), PR (*d* = 1.94; *p* = 0.007), and TR (*d* = 3.03; *p* < 0.001) groups.

**TABLE 1:** Changes in antioxidants parameters following RT with and without blood flow restriction.

	**Week 0**		**Week 8**	
	**Pre-exercise**	**Post-exercise**	**Pre-exercise**	**Post-exercise**
**FRAP**				

Traditional	1.56 ± 0.21	0.58 ± 0.21*^#^	1.30 ± 0.44	0.70 ± 0.31*^#^
Partial restriction	1.22 ± 0.40	1.02 ± 0.34	1.32 ± 0.35	1.07 ± 0.26
Total restriction	1.29 ± 0.31	0.64 ± 0.22*^#^	1.40 ± 0.33	0.82 ± 0.31*

**GSH**				

Traditional	3.39 ± 0.91	2.15 ± 1.39	3.97 ± 1.91	2.38 ± 1.55*
Partial restriction	3.70 ± 1.46	3.27 ± 2.32	3.80 ± 1.93	3.10 ± 1.24
Total restriction	3.99 ± 3.22	1.85 ± 2.02*	4.65 ± 1.24	2.29 ± 1.08*

**GSSG**				

Traditional	7.18 ± 0.96*	10.22 ± 1.13	10.18 ± 1.86	14.20 ± 3.38*
Partial restriction	9.63 ± 1.66	11.01 ± 1.94	10.82 ± 2.01	11.25 ± 1.72
Total restriction	8.73 ± 1.21*	12.51 ± 2.88	11.18 ± 1.68	15.78 ± 2.68*^#&^

**GSH/GSSG ratio**				

Traditional	0.56 ± 0.17*	0.21 ± 0.14	0.39 ± 0.18	0.16 ± 0.08*
Partial restriction	0.37 ± 0.08	0.29 ± 0.17	0.36 ± 0.21	0.28 ± 0.12
Total restriction	0.46 ± 0.38*	0.14 ± 0.12	0.41 ± 0.10	0.15 ± 0.08*

Results are presented as mean ± SD. FRAP, plasma iron reduction ability; GSH, reduced glutathione; GSSG, oxidized glutathione content. ^*^p < 0.05 from pre-exercise; ^#^p < 0.05 from PR; and ^&^p < 0.05 from post-exercise at week 0.

Glutathione results showed an effect of time (*p* < 0.001; η^2^_*p*_ = 0.25), with no difference among groups (*p* = 0.79; η^2^_*p*_ = 0.02) or time × group interaction (*p* = 0.42; η^2^_*p*_ = 0.08). Compared to pre-exercise, GSH was reduced immediately after exercise in response to TR at week 0 (*d* = 0.94; *p* = 0.02), but not in TRAD (*p* = 0.08) or PR (*p* = 1.00). At week 8, GSH was reduced after exercise in response to TRAD (*d* = 0.99; *p* = 0.007) and TR (*d* = 1.75; *p* < 0.001).

At baseline, there was a difference among groups for GSSG (*p* = 0.002; η^2^_*p*_ = 0.40), and pairwise comparisons revealed higher concentration of GSSG for the PR (*d* = 1.80; *p* = 0.002) when compared to TRAD. Therefore, analysis of covariance was used for time and between group comparisons. Compared to pre-exercise, there was an increase in GSSG after exercise (*p* = 0.028; η^2^_*p*_ = 0.14), and results showed a significant effect of group (*p* = 0.002; η^2^_*p*_ = 0.46) and time × group interaction (*p* < 0.001; η^2^_*p*_ = 0.36). At week 0, GSSG concentration was higher after exercise for the TRAD (*d* = 2.75; *p* = 0.02) and TR (*d* = 1.38; *p* < 0.001) groups, but not for the PR (*p* = 0.07) group. At week 8, GSSG concentration was increased after exercise in the TRAD (*d* = 1.66; *p* = 0.003) and TR (*d* = 1.88; *p* < 0.001) groups. Compared to week 0, post-exercise levels of GSSG at week 8 were significantly higher in response to TR (*d* = 1.01; *p* = 0.03).

There was a decrease after exercise in GSH/GSSG ratio (*p* < 0.001; η^2^_*p*_ = 0.43), with no difference among conditions (*p* = 0.72; η^2^_*p*_ = 0.03) or time × group interaction (*p* = 0.055; η^2^_*p*_ = 0.15). At week 0, GSH/GSSG ratio was reduced after exercise in the TRAD (*d* = 2.63; *p* = 0.001) and TR (*d* = 0.95; *p* = 0.009) groups, but not in PR (*p* = 1.00) group.

### Oxidative Stress Biomarkers

Table 2 shows the acute responses of TBARS, thiols, and protein carbonyls in response to RT. There was an increase in TBARS after exercise (*p* < 0.001; η^2^_*p*_ = 0.61) and a time × group interaction (*p* < 0.001; η^2^_*p*_ = 0.31), with no significant difference (main effect) among groups (*p* = 0.19; η^2^_*p*_ = 0.13). At week 0, there was an increase in TBARS after exercise for the TRAD (*d* = 1.46; *p* < 0.001) and TR (*d* = 2.04; *p* < 0.001) groups, but not for the PR (*p* = 1.00) group. At week 8, TBARS was higher after exercise in TRAD (*d* = 2.15; *p* < 0.001) and TR (*d* = 2.21; *p* < 0.001).

**TABLE 2:** Changes in oxidative stress biomarkers following RT with and without blood flow restriction.

	**Week 0**		**Week 8**	
	**Pre-exercise**	**Post-exercise**	**Pre-exercise**	**Post-exercise**
**TBARS**				

Traditional	55.4 ± 8.48	88.4 ± 23.96*^#^	52.3 ± 5.63	92.2 ± 22.70*
Partial restriction	52.4 ± 11.77	61.3 ± 12.05	67.7 ± 20.12	74.1 ± 19.16
Total restriction	50.8 ± 13.54	86.4 ± 16.34*^#^	54.8 ± 15.05	96.7 ± 14.37*

**Thiols**				

Traditional	35.6 ± 5.10	29.2 ± 6.29	33.7 ± 12.69	31.2 ± 10.53
Partial restriction	34.3 ± 2.80	32.1 ± 3.49	35.1 ± 7.34	30.5 ± 5.26
Total restriction	41.7 ± 11.30	35.8 ± 8.80	42.1 ± 9.90	37.7 ± 13.02

**Protein carbonyls**				

Traditional	83.1 ± 8.82	125.6 ± 15.44*^#^	83.0 ± 8.60	116.5 ± 18.18*^#^
Partial restriction	82.6 ± 4.30	87.4 ± 6.53	82.7 ± 4.79	86.6 ± 5.31
Total restriction	79.7 ± 3.34	103.1 ± 13.35*^#^	80.8 ± 8.83	120.3 ± 13.56*^#&^

Results are presented as mean ± SD. *Different from pre-exercise p < 0.05; ^#^different from PR group p < 0.05; and ^&^different from post-exercise at week 0 *p* < 0.05.

There was no significant effect of RT on thiol groups after exercise (*p* = 0.07; η^2^_*p*_ = 0.11) and no time × group interaction (*p* = 0.92; η^2^_*p*_ = 0.02).

For protein carbonyl, there was a significant difference across time (*p* < 0.001; η^2^_*p*_ = 0.79) among groups (*p* < 0.001; η^2^_*p*_ = 0.48) and a time × group interaction (*p* < 0.001; η^2^_*p*_ = 0.62) was revealed. At week 0 compared to pre-exercise, there was an increase in protein carbonyls after exercise for the TRAD (*d* = 4.06; *p* < 0.001) and TR (*d* = 1.87; *p* < 0.001) groups, but not for the PR (*p* = 0.89) group. At week 8, protein carbonyls increased after exercise in the TRAD (*d* = 2.90; *p* < 0.001) and TR (*d* = 4.35; *p* < 0.001) groups. Compared to week 0, post-exercise levels of protein carbonyls were significantly higher at week 8 in response to TR (*d* = 1.11; *p* = 0.008).

### Caspase-3 Activity in Neutrophils and Lymphocytes

The viability of neutrophils ranged from 97.4 to 99.3% and from 96.3 to 99.2% at week 0 and week 8, respectively (data are not shown). The viability of lymphocytes ranged from 94.4 to 97.9% and from 95.2 to 98.2% at week 0 and week 8, respectively (data are not shown).

**FIGURE 3:**
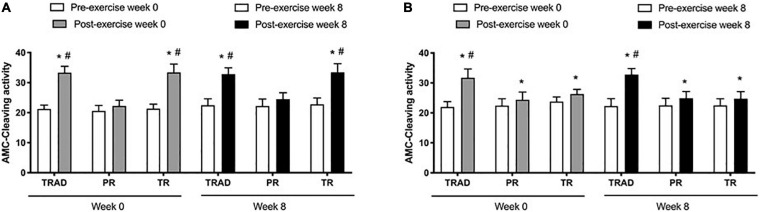
Caspase-3 activity in neutrophils **(A)** and lymphocytes **(B)** using in 5 × 10^5^ cells following RT with and without BFR. TRAD, traditional resistance training; PR, partial restriction; TR, total restriction. Results are presented as mean ± SD. *Compared to pre-exercise and ^#^compared to PR.

There was an increase in caspase-3 activity in neutrophils after exercise (*p* < 0.001; η^2^_*p*_ = 0.87) versus pre-exercise, with a significant difference among groups (*p* < 0.001; η^2^_*p*_ = 0.80) and time × group interaction (*p* < 0.001; η^2^_*p*_ = 0.66). At week 0, there was an increase in caspase-3 levels after exercise for the TRAD (*d* = 6.31; *p* < 0.001) and TR (*d* = 4.60; *p* < 0.001) groups, but not in the PR (*p* = 0.10) group. At week 8, caspase-3 increased after exercise in the TRAD (*d* = 4.57; *p* < 0.001) and TR (*d* = 3.22; *p* < 0.001) groups. There was a greater increase in caspase-3 activity after exercise in the TRAD (*d* = 5.55; *p* < 0.001) and TR (*d* = 4.74; *p* < 0.001) groups versus the PR group at week 0. At week 8, caspase-3 levels were higher in the TRAD (*d* = 3.95; *p* < 0.001) and TR (*d* = 3.55; *p* < 0.001) groups when compared with the PR group (Figure 3A).

Results exhibited an increase in lymphocytes after exercise (*p* < 0.001; η^2^_*p*_ = 0.74), with significant difference between groups (*p* < 0.001; η^2^_*p*_ = 0.53) and a time × group interaction (*p* < 0.001; η^2^_*p*_ = 0.63). At week 0, there was an increase in caspase-3 levels after exercise for the TRAD (*d* = 3.87; *p* < 0.001), PR (*d* = 2.38; *p* = 0.006), and TR (*d* = 4.72; *p* < 0.001) groups. At week 8, caspase-3 increased after exercise in the TRAD (*d* = 4.53; *p* < 0.001), PR (*d* = 1.75; *p* = 0.01), and TR (*d* = 1.99; *p* < 0.001) groups. There was a greater increase in caspase-3 levels after exercise in the TRAD group when compared with the PR group at week 0 (*d* = 2.71; *p* < 0.001) and at week 8 (*d* = 3.73; *p* < 0.001) (Figure 3B).

### Mitochondrial Membrane Potential

**FIGURE 4:**
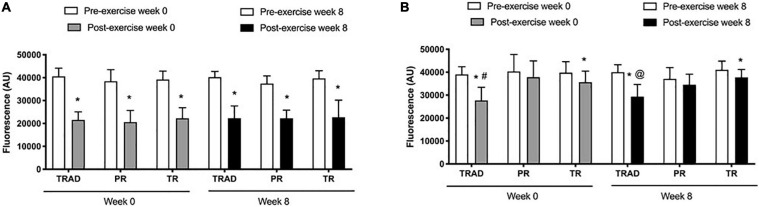
Mitochondrial membrane potential in neutrophils **(A)** and lymphocytes **(B)** using in 5 × 10^5^ cells following RT with and without BFR. TRAD, traditional resistance training; PR, partial restriction; TR, total restriction. Results are presented as mean ± SD. *Compared to pre-exercise, ^#^compared to PR and TR, and ^@^compared to TR.

Compared to pre-exercise, there was a decrease in MMP in neutrophils after exercise (*p* < 0.001; η^2^_*p*_ = 0.86), but no difference among groups (*p* = 0.38; η^2^_*p*_ = 0.08) or time × group interaction (*p* = 0.96; η^2^_*p*_ = 0.02). At week 0, MMP was lower after exercise in the TRAD (*d* = 4.83; *p* < 0.001), PR (*d* = 2.60; *p* < 0.001), and TR (*d* = 4.40; *p* < 0.001) groups. At week 8, MMP was reduced after exercise in response to TRAD (*d* = 2.13; *p* < 0.001), PR (*d* = 3.42; *p* < 0.001), and TR (*d* = 2.85; *p* < 0.001) (Figure 4A).

Results revealed a reduction of MMP in lymphocytes after exercise (*p* < 0.001; η^2^_*p*_ = 0.36), with a significant difference among groups (*p* = 0.01; η^2^_*p*_ = 0.31) and time × group interaction (*p* = 0.01; η^2^_*p*_ = 0.25). At week 0, there was a decrease of MMP after exercise in the TRAD (*d* = 2.04; *p* < 0.001) and TR (*d* = 1.39; *p* = 0.02) groups, but not in PR (*p* = 0.41) group. At week 8, MMP decreased after exercise in the TRAD (*d* = 2.27; *p* < 0.001) and TR (*d* = 1.39; *p* = 0.03) groups. At week 0, there was a greater decrease of MMP in lymphocytes after exercise in response to TRAD versus TR (*d* = 1.50; *p* = 0.004) and PR (*d* = 1.57; *p* = 0.03). At week 8, MMP was lower in the TRAD when compared with the TR (*d* = 1.89; *p* = 0.002) group (Figure 4B).

## Discussion

The hypothesis of our study was met in which the indices of oxidative stress (reduction in FRAP, GSH, and GSH/GSSG ratio and increase in GSSG, TBARS, and protein carbonyls) and leukocyte apoptosis (decrease in MMP and increase in caspase-3 activity) were greater after exercise in the high-load RT and with total BFR versus pre-exercise. However, acute RT did not significantly increase blood oxidative stress after exercise or elicit lower markers of apoptosis in adults undergoing RT with partial restriction. Moreover, improvements in muscular strength in response to high-load RT and BFR were similar, suggesting that partial BFR training can be used as an alternative training method to improve muscular strength.

Our results revealed that low-load RT with partial BFR did not increase biomarkers of oxidative stress post-exercise. Neto et al. (2018) compared the effects of continuous and intermittent upper body resistance exercises with partial BFR (20% 1-RM) on changes in protein carbonyls and TBARS in resistance-trained men. The authors reported no significant increase in biomarkers of oxidative stress after exercise in both protocols. Previous studies in resistance-trained men confirmed these findings demonstrating no significant changes in TBARS and protein carbonyls following arm curl resistance exercise with partial BFR (Goldfarb et al., 2008; Garten et al., 2015). Therefore, although low-load RT with partial BFR promotes an increase in the production of ROS (Centner et al., 2018), this increase seems inadequate to cause damage to macromolecules, and it may reflect a normal physiological response to exercise.

In contrast, high-load and low-load RT with total BFR promoted large to very large changes in oxidative stress biomarkers immediately post-exercise at baseline and after 8 weeks of training. Our results indicated that TRAD and total BFR similarly increased TBARS, protein carbonyls, concentration of GSSG, and decreased GSSG/GSH and FRAP after exercise. In resistance-trained men, Goldfarb et al. (2008) demonstrated an increase in protein carbonyls and GSSG/GSH ratio after arm curl resistance exercise (70% 1-RM) to failure, while no change was observed with a partial BFR protocol (30% 1-RM). Evidence indicates that moderate to high-load resistance exercise can acutely elevate biomarkers of oxidative stress (Garten et al., 2015; da Silva et al., 2016). Our results reveal that performing RT with total BFR may elicit comparable metabolic disturbance and ROS generation during exercise versus high-load RT (Suga et al., 2012; Kim et al., 2014) despite the lower external load. It has been suggested that decreasing PO_2_ increases the generation of ROS from xanthine oxidase during high-intensity (Hellsten et al., 1997; Goldfarb et al., 2008) and BFR exercise (Petrick et al., 2019). The variation of the cuff pressure may also interfere with the oxygen availability during exercise, in turn altering the magnitude of hypoxia and hyperoxia and consequently modifying the magnitude of potential oxidative stress (Ganesan et al., 2015).

In disagreement with our hypothesis, there was no change in TBARS, protein carbonyls, FRAP, GSH, or GSSG/GSH ratio after 8 weeks of traditional RT. However, some prior work indicated a decrease (Alikhani and Sheikholeslami-Vatani, 2019) while others have reported no change (Bobeuf et al., 2011) or increase in oxidative stress biomarkers after chronic RT (Gargallo et al., 2018). A likely explanation for these discrepant findings is methodological differences between studies including discrepancies in the training status of participants as well as inadequate control of confounding factors such as acute dietary intake and physical activity pre-exercise.

A moderate level of ROS production during exercise stimulates metabolic adaptation in different cells and tissues, while high levels of ROS production elicit damage to proteins, lipids, and DNA (Powers et al., 2020). Still, it appears unlikely that high-load RT and RT with total BRF amplifies oxidative stress sufficiently to be detrimental to human health and performance. Our results indicate no improvements in blood antioxidant capacity or change in GSH and GSSG/GSH ratio after 8 weeks of RT with or without BFR. In agreement with our data, Carru et al. (2018) reported no changes in total antioxidant capacity after 18 weeks of RT at 70% of 1-RM in untrained older subjects. Similarly, there was no increase in total blood antioxidant capacity after 6 months of RT at 80% 1-RM in untrained young men and women (Bobeuf et al., 2011). In addition, Gargallo et al. (2018) showed that 16 weeks of high-intensity RT with elastic bands increased oxidative stress, as shown by increased DNA damage and a decrease in GSSG/GSH ratio in older women.

Our findings show a very large acute response of biomarkers of apoptosis in leukocytes immediately after exercise in response to TRAD and total BFR at baseline and after 8 weeks of RT. We found a reduction of MMP in lymphocytes after exercise in response to TRAD and TR, suggesting that high-load traditional RT and total BFR promote activation of intrinsic pathway of lymphocyte apoptosis. Results from Krüger et al. (2011) in trained men showed a decrease of MMP in lymphocytes after a session of whole-body RT at 75% of 1-RM, but not after a moderate intensity RT protocol (60% 1-RM). Our findings are in agreement with Krüger et al. (2011) indicating that low-load RT with total BFR elicited a similar pattern of lymphocytes response after exercise compared to high-load traditional RT, despite the differences in exercise intensity (20 versus 75% 1-RM, respectively). Therefore, RT performed with total arterial occlusion pressure to failure can induce localized hypoxia and metabolic stress (Willis et al., 2019) and increase apoptosis signaling in leukocytes (Guo et al., 2015). Moreover, there was no change in MMP in lymphocytes after exercise in response to PR, indicating that the effect of low-load RT with partial BRF on MMP in lymphocytes may be comparable to moderate intensity RT.

Prior studies observed that the acute response of markers of apoptosis to RT depends on exercise intensity (Krüger et al., 2011; Pereira et al., 2012; Prestes et al., 2014). Prestes et al. (2014) reported a significant increase in lymphocyte apoptosis immediately after RT at 10-repetitions maximum (10-RM) (100% of 10-RM) compared to a lighter load (60% of 10-RM) in untrained young adults. It is plausible that at higher exercise intensities, substantially greater cellular migration occurs which removes the apoptotic cells from vasculature.

Mitochondria play a central role in regulation of the intrinsic apoptosis pathway (Otton and Curi, 2005). After the permeabilization of the mitochondrial membrane, caspases-3, -6, and -7 are activated, resulting in the mediation and amplification of the death signal (Kruger and Mooren, 2014). Our results show a significant (large “effect”) increase in caspase-3 activity after exercise in lymphocytes and neutrophils in response to TRAD and TR, with no difference seen between baseline and after 8 weeks of RT. Our findings corroborate results from Sharafi and Rahimi (2012) who documented an acute increase in blood caspase-3 activity immediately and 3 h post-resistance exercise at 80% 1-RM in untrained men. Moreover, Yang et al. (2006) reported an increase in caspase-3 mRNA expression after lower body resistance exercise at 65% 1-RM in untrained men. Overall, these findings confirm the onset of exercise-induced apoptosis in different cell types in response to RT (Mooren et al., 2004; Syu et al., 2011; Sharafi and Rahimi, 2012; Kruger and Mooren, 2014; Prestes et al., 2014).

Our data (Figure 4) show no change in MMP of lymphocytes post-exercise in response to PR. The exercise-induced increase in caspase-3 activity may have been activated by other upstream factors rather than the intrinsic apoptosis pathway mediated by the reduction of MMP. In addition, caspase-3 is also involved in other cellular functions besides the apoptosis cascade. For example, caspase-3 participates during the early stages of lymphocytes activation and proliferation in the absence of detectable cell death (Miossec et al., 1997). Overall, our data emphasize that low-load RT with partial BFR promotes low level of oxidative stress, and subsequent lower biomarkers of leukocyte apoptosis compared to traditional RT and total restriction.

Our data exhibit similar strength gains in response to traditional high-load RT (15%) and low-load RT with partial (16%) and total BFR (14%). Our data are supported by a recent meta-analysis suggested by Gronfeldt et al. (2020), which demonstrated no difference between traditional high-load (60–90% 1-RM) and low-load (20–30% 1-RM) RT with BFR on changes in muscle strength in untrained men. This similar increase in strength is likely due to the effect of BFR in inducing neural adaptations to RT (motor unit recruitment, firing rate, and synchronization). Centner and Lauber (2020) reported that low-load RT with BFR promoted significantly enhanced levels of muscle excitation compared to traditional low-load RT. Moreover, compared to high-load RT, muscle excitation following low-load RT with BFR was similar or slightly lower. Our results suggest no effect of cuff pressure on strength gains, which reveals that different pressures used to employ BFR during low load RT do not seem to induce discrepant changes in muscle strength in untrained adults.

This study has a few limitations. First, all participants were young men and hence our results are not applicable to different populations including women, the elderly, and resistance-trained subjects. Furthermore, we did not measure changes in nutritional intake throughout the study, although men were asked to maintain their nutritional habits. In addition, we used the arm curl exercise because it is a very common resistance exercise, although different results may be revealed with RT using a larger muscle mass of the lower extremity.

## Conclusion

In summary, data show that low-load RT with partial BFR can be a potent stimulus to increase muscular strength but still does not augment biomarkers of oxidative stress in young untrained men. In addition, RT with total BFR promoted similar responses of oxidative stress and markers of neutrophils and lymphocyte apoptosis versus high-load traditional RT. Additional research is needed to examine how gender, training status, age, and training frequency and duration can influence the chronic adaptations in biomarkers of leukocyte apoptosis and oxidative stress in response to RT.

Based on our current results, practitioners and exercise professionals may consider implementing RT with partial BFR as an effective training method for the development of muscular strength in exercise and rehabilitation programs, while minimizing metabolic stress and the negative impact on the immune response.

## Data Availability Statement

The raw data supporting the conclusions of this article will be made available by the authors, without undue reservation.

## Ethics Statement

This study was approved by the Ethics Committee of Cruzeiro do Sul University under protocol 64377817.4.0000.8084. The patients/participants provided their written informed consent to participate in this study.

## Author Contributions

FL and RO participated in the design of the study and contributed to the data collection and data analysis. LF and CS contributed to the data collection and data analysis. DM, TA, JP, and MM contributed to the data analysis and interpretation of the results. All authors contributed to the article and approved the submitted version.

## Conflict of Interest

The authors declare that the research was conducted in the absence of any commercial or financial relationships that could be construed as a potential conflict of interest.

## Publisher’s Note

All claims expressed in this article are solely those of the authors and do not necessarily represent those of their affiliated organizations, or those of the publisher, the editors and the reviewers. Any product that may be evaluated in this article, or claim that may be made by its manufacturer, is not guaranteed or endorsed by the publisher.
